# SCA-1/Ly6A Mesodermal Skeletal Progenitor Subpopulations Reveal Differential Commitment of Early Limb Bud Cells

**DOI:** 10.3389/fcell.2021.656999

**Published:** 2021-07-16

**Authors:** Jessica Cristina Marín-Llera, Carlos Ignacio Lorda-Diez, Juan Mario Hurle, Jesús Chimal-Monroy

**Affiliations:** ^1^Departamento de Medicina Genómica y Toxicología Ambiental, Instituto de Investigaciones Biomédicas, Universidad Nacional Autónoma de México, México, México; ^2^Departamento de Anatomía y Biología Celular and IDIVAL, Facultad de Medicina, Universidad de Cantabria, Santander, Spain

**Keywords:** progenitor cell, limb bud, SCA-1/Ly6A, tenogenic differentiation, chondrogenesis, recombinant limbs

## Abstract

At early developmental stages, limb bud mesodermal undifferentiated cells are morphologically indistinguishable. Although the identification of several mesodermal skeletal progenitor cell populations has been recognized, in advanced stages of limb development here we identified and characterized the differentiation hierarchy of two new early limb bud subpopulations of skeletal progenitors defined by the differential expression of the SCA-1 marker. Based on tissue localization of the mesenchymal stromal cell-associated markers (MSC-am) CD29, Sca-1, CD44, CD105, CD90, and CD73, we identified, by multiparametric analysis, the presence of cell subpopulations in the limb bud capable of responding to inductive signals differentially, namely, sSca^+^ and sSca^–^ cells. In concordance with its gene expression profile, cell cultures of the sSca^+^ subpopulation showed higher osteogenic but lower chondrogenic capacity than those of sSca^–^. Interestingly, under high-density conditions, fibroblast-like cells in the sSca^+^ subpopulation were abundant. Gain-of-function employing micromass cultures and the recombinant limb assay showed that SCA-1 expression promoted tenogenic differentiation, whereas chondrogenesis is delayed. This model represents a system to determine cell differentiation and morphogenesis of different cell subpopulations in similar conditions like *in vivo*. Our results suggest that the limb bud is composed of a heterogeneous population of progenitors that respond differently to local differentiation inductive signals in the early stages of development, where SCA-1 expression may play a permissive role during cell fate.

## Introduction

In the early stages of development, limb mesoderm is a histologically homogeneous tissue composed of progenitor cells that originates most adult limb tissues (Zwilling, 1961). How mesodermal skeletal progenitors (MSP) differentiate into chondrogenic, osteogenic, and tenogenic lineages, what signals are present in the early limb bud, and how cells pattern to a functional limb have been widely studied (reviewed by Tickle, 2001; Marín-Llera et al., 2019). Since the early stages of limb development, the mesodermal cells receive different signals that commit cells to distinct cell fates. Although mesodermal cells seem histologically homogenous, they molecularly become different in response to the limb patterning signals released from the three signaling centers of the limb. Thus, establishing the spatial pattern of limb tissues results from the fine-tune activation of the molecular machinery triggered by specific transcription factors for each lineage. Among the transcription factors involved in chondrogenic and tenogenic differentiation are SRY-Box Transcription Factor 9 (*Sox9*) and the bHLH *Scleraxis* (*Scx*) (de Crombrugghe et al., 2000; Schweitzer et al., 2001; Akiyama et al., 2002). Once the cartilaginous skeletal primordia are formed in the core of the limb, endochondral ossification is initiated and the early osteoprogenitors express the Runt Related Transcription Factor 2 (*Runx2*) (Chen et al., 2014). However, it is unknown whether the mesodermal progenitors of the early limb respond differently to the same inductive signals because of the presence of different committed cell subpopulations. Alternatively, those cell subpopulations may represent multipotent progenitors. A first approximation by Pearse et al. (2007) determined the clonal relationship of limb cells in the early chick embryo and identified the final tissues where clones integrated. Lentiviral injections at three developing stages (16HH, 18HH, and 20HH) showed a frequent dual lineage contribution in adjacent tissues. The authors concluded that as development advances, the number of such multilineage clones is reduced. Furthermore, they observed that the size of the clone (the number of cells descended from each infected cell) decreased as development progressed, suggesting the existence of a multipotent cell population in the early limb bud (Pearse et al., 2007). However, they did not demonstrate the presence of specific committed cell subpopulations with different differentiation potentials. One limitation of that work is the lack of specific markers to isolate and characterize different populations originating in each lineage.

A strategy that allows for identifying limb cell populations consists of multiparametric analysis using markers associated with multipotent mesenchymal stromal cells (MSCs). MSC can differentiate into mesodermal tissues, including limb tissues such as bone, cartilage, tendon, ligament, dermis, and muscle, among others (reviewed by Caplan, 1991; Marquez-Curtis et al., 2015). Besides plastic adherence, positive expression of the surface markers CD29, CD73, CD90, CD105, CD44, and negative expression for CD45, CD34, CD19, CD11b, CD79α, and HLA-DR are necessary to identify MSC (Dominici et al., 2006). The MSC-associated markers (MSC-am) allow the identification of cell subpopulations. *In vitro*, cells acquire MSC-am and they may not represent in vivo populations (Boiret et al., 2005; Bara et al., 2014; Guimarães-Camboa et al., 2017; Marín-Llera and Chimal-Monroy, 2018). However, few works has been focus on identifying cell subpopulations without a previous culture (Jones et al., 2006; Chan et al., 2015; Reinhardt et al., 2019). In the adult mouse limb, subpopulations with specific signatures are recognized; the mouse skeletal stem cell (mSSC) population (CD45^–^, Ter-119^–^, Tie2^–^, AlphaV^+^, Thy^–^, 6C3^–^, CD105^–^, and CD200^+^) identified by Chan et al. (2015); the PαCD51 (PDGFRa^+^, CD51^+^) identified by Pinho et al. (2013), and the PαS (PDGFRa^+^, Sca-1^+^, CD45^–^, TER119^–^) determined by Morikawa et al. (2009). Notably, although these works are an antecedent in the use of MSC-am to identify limb subpopulations, these subpopulations have been characterized only in adult limbs or in advanced stages of limb development. In contrast, at the early stages of mouse development, Reinhardt et al. (2019) identified three distinct cell populations in the posterior distal mesoderm and the core and peripheral mesoderm of mouse forelimbs. The subpopulations SOX9^–^, JAG^+^, Lin^–^ and SOX9^–^, PDGFRα^*hi*^, SCA^–^, JAG^–^, Lin^–^ were identified as immature progenitors, SOX9^–^, PDGFRα^*hi*^, SCA^+^, JAG^–^, Lin^–^ as myogenic progenitors while the SOX9^+^, PDGFRα^*hi*^, Lin^–^ represent osteochondro progenitors (OCP) (Reinhardt et al., 2019).

In the present study, we determined the tissue localization of the CD29, Sca-1, CD44, CD105, CD90, and CD73 MSC-am in the E10.5 mouse limb bud and, based on its pattern expression. we identified and characterized limb MSP subpopulations at early stages of development without previous *in vitro* expansion. Our study provides evidence for the presence of two subpopulations based on a combination of MSC-am and SCA-1 expression in the limb bud. SCA-1 may be related to establishing differential commitment stages or the maintenance of the competence for osteogenic and tenogenic cell fates but not for chondrogenic fate in the early limb bud. In conclusion, we identified and characterized two novel limb MSP subpopulations at the early stages of development.

## Materials and Methods

### Ethics

The studies involving animals were reviewed and approved by the Institutional Review Board for the Care and Use of Laboratory Animals of the Instituto de Investigaciones Biomédicas, Universidad Nacional Autónoma de México (UNAM, Mexico City, Mexico). Mice were obtained from the animal facility of the Instituto de Investigaciones Biomédicas, UNAM. All procedures were performed according to the guidelines for the Institutional Review Board for the Care and Use of Laboratory Animals of the Instituto de Investigaciones Biomédicas, UNAM.

### Sample Obtaining

#### Mice Embryo Hindlimbs Obtaining

CD-1 strain pregnant mice at 10.5 days post coitum (E10.5), 11.5 days post coitum (E11.5), and 12.5 days post coitum (E12.5) were killed by CO_2_ asphyxia. Embryos were removed from the uterus and handled, according to Marín-Llera and Chimal-Monroy (2018). For each experiment, a different number of pregnant mice with approximately ten embryos each was required: three-pregnant mice for each sample acquisition to determine the percentage of sSca^+^ and sSca^–^ subpopulations by flow cytometry (∼60 hindlimbs) and 10- to 12-pregnant mice for each experiment of subpopulations cell sorting (∼200–240 hindlimbs). For all flow cytometry acquisition analyses and Fluorescence-Activated Cell Sorting (FACS), hindlimb embryo cells were incubated for 5 min in AKC lysis buffer (150 mM NH_4_Cl, 10 mM KHCO_3_, 0.1 mM Na_2_EDTA at pH 7.4) and resuspended in FACS buffer (5 U/ml DNAsa, 1 mM EDTA, 1% of inactivated fetal bovine serum (FBS) (Life Technologies) and 25 mM HEPES diluted in free Ca^++^ and Mg^++^ solution at pH 7.4) for antibody staining. For *in vitro* differentiation, electroporation assays, and recombinant limbs (RLs), hindlimb cells were resuspended in DMEM-HG medium (Life Technologies, Carlsbad, CA, United States) until their use.

#### Cell Suspensions From Hindlimbs Chick Embryos

Fertilized White Leghorn chicken eggs (ALPES, Puebla, Mexico) were incubated at 38°C and staged according to Hamburger and Hamilton (1951). The eggs were windowed at stage 22–23HH; embryos were removed from the egg and washed in PBS 1×. Whole hindlimb buds were dissected out and dissociated with 2 mg/mL collagenase type IV (Life Technologies) in Hanks Solution at 37°C for 5 min. Limb buds were resuspended in DMEM-HG medium supplemented with 10% of FBS (Life Technologies) to inactivate collagenase and pipetted until a single-cell suspension was obtained. The cell suspension was centrifuged at 1,100 rpm for 5 min. After obtention, cells were resuspended in DMEM-HG medium (Life Technologies).

### Flow Cytometry and Cell Sorting

For sample acquisition to determine the percentage of sSca^+^ and sSca^–^ subpopulations in E10.5, E11.5, and E12.5 hindlimb cells, one million mouse limb bud cells were resuspended in 100 μL of FACS buffer containing a mixture of CD29-APC (1:100; cat. 102215), Sca-1-PE/Cy7 (1:300; cat. 108113), CD44-FITC (1:300; cat.103007), CD117-APC/Cy7 (1:100; cat 105825), and CD45-PerCP/Cy5 (1:500; cat. 103131), all from BioLegend (San Diego, CA, United States), and PE anti-mouse Flk1 (1:300, cat. no. 555308, BD Pharmigen, San Jose, CA, United States) antibodies. The cell suspension was incubated for 30 min on ice protected from light. Unlabeled cells and isotype antibodies were used as controls to exclude non-specific fluorescence. For cell sorting, between fifteen and twenty million mouse hindlimb bud cells were stained for each experiment. Sample acquisition was performed using a flow cytometer, Attune NXT (Life Technologies), and sorting was done using FACS Aria II (BD Biosciences, San Jose, CA, United States), with at least 20,000 events being collected. Data were analyzed using FlowJo Software version 10. Single cells were selected from the dot plot of side scatters height and area, and gates were determined by the FMO isotype control to scatter. Each acquisition and sorting experiments were run in triplicate and represented a pool of approximately 60 and 200 hindlimb buds, respectively.

### *In vitro* Differentiation Assays

For osteogenic differentiation, mouse limb subpopulations and total cells were directly sorted at 30,000 cells/cm^2^ in 96 well plates to reach the 80% confluence. Cells for each condition were incubated for 15 days in 1 mL of Complete MesenCult Osteogenic Medium (StemCell Technologies, Vancouver, BC, Canada). Debris or detached cells were washed with PBS, and the medium was replaced with fresh medium every 3–4 days. After 15 days of induction, differentiated cells were washed with PBS and fixed in 4% paraformaldehyde for 30 min at 4°C. For alizarin red staining, cells were fixed in 10% of formaldehyde-PBS for 10 min at room temperature. After washing, cells were stained with 0.2% Alizarin S-Red solution (pH 4.2) for 20 min. Excess of alizarin staining was washed twice with water for further image acquisition.

Chondrogenic differentiation was evaluated by micromass assays. Freshly isolated subpopulations and total cells were directly seeded in 48 well plates at 3 × 10^5^ cells in 10 μL of DMEM-HG medium (Life Technologies), supplemented with 10% FBS (Life Technologies). After permit cell attachment for 2 h, micromass was flooded in DMEM-HG medium (Life Technologies). Cultures were maintained for 3 days under a 5% CO_2_ atmosphere until Alcian blue staining. Chondrogenic and osteogenic differentiation images were acquired with an AxioZoom V.16 fluorescence microscope (Carl Zeiss).

### Electroporation Assays

For each electroporation, 15 × 10^6^ chick hindlimb cells were obtained from ∼30 freshly isolated 22–23HH embryos (∼60 hindlimbs). Immediately after, 5 × 10^6^ cells were resuspended in Multiporator Electroporation Buffer Isosmolar (Eppendorf, Hamburg, Germany, cat. no. EMP4308070510), transferred into cuvettes, and electroporated with 1.5 μg of *Sca-1*-GFP (Origene, cat. no. MG200790) or GFP (sham) plasmid per million cells using an Eppendorf Multiporator System. Non-electroporated cells were handled as a sham and *Sca1* electroporated cells. Immediately after their electroporation, cells for each condition were resuspended in DMEM-HG medium (Life Technologies), supplemented with 10% FBS (Life Technologies). Uncultured cells were centrifugated for 5 min at 1100 rpm and incubated at 37°C for 2 h to form a pellet for RLs or seeded for micromass assays to evaluate gene regulation.

### Micromass Culture

Chick limb bud cells were plated for triplicate in 48 well plates at a density of 3 × 10^5^ cells/10 uL in the DMEM-HG medium (Life Technologies) according to Ahrens et al. (1977) and supplemented with 10% of FBS (Life Technologies), 100 U/mL penicillin and 100 mg/mL streptomycin (Sigma-Aldrich), 1× non-essential amino acids (Life Technologies), and 1× GlutaMAX (Life Technologies) in a humidified incubator containing 5% CO_2_ at 37°C for 2 h. Posteriorly, micromass were flooded in DMEM-HG medium without serum. After 72 h of incubation, the micromass were fixed in Khale’s fixative for Alcian blue staining or in 4% PFA for *in situ* hybridization. All micromass assays were performed by triplicate.

### Real-Time RT-PCR

RNA extractions were performed with NucleoSpin RNA (Macherey-Nagel, cat. no. 740955, Düren, Germany) according to the manufacturer’s instructions. Retrotranscription of total RNA was performed using the RevertAid RT kit (Thermo Fisher Scientific, cat. no. K1691, Waltham, MA, United States). Expression levels were analyzed using a real-time PCR system and quantified with SYBR green (Thermo Fisher). The *Gapdh* gene was used as a normalizer and a melting curve to analyze the specificity of the amplification. The expression level was evaluated relative to a calibrator according to the 2^–(ΔΔ*Ct)*^ equation. Each value represented the mean ± SEM of at least three independent experiments and was analyzed using Student’s *t*-test. Statistical significance was set at *p* < 0.05. The sequences of primers used in this study are included in Table 1.

**TABLE 1:** Oligonucleotide sequences employed for qRT-PCR.

Gene	Forward (5′-3′)	Reverse (5′-3′)
*Prx1*	tctcagagtggttgactctctcc	gaactaccatcacgctctctcc
*Msx1*	cctacgcaagcacaagacc	tcggcaatagacaggtactgc
*Ap2*	cctcgaagtacaaggtcacg	cgacgcgttaagacactcg
*Spouty 1*	agcctgctacgattctgtcc	gagcaggtcttctcgctacc
*Sprouty 2*	ggtctcggagcagtacaagg	cactcggattattccatcagc
*Meis 1*	atgatgcatggaggacagc	tgacttgtgcactcattgtcg
*Sox9*	ctgaagaaggagagcgagga	gtccagtcgtagcccttcag
*Scx*	aacacggccttcactgc	gcagcgtctcaatcttgg
*Runx2*	gactgtggttaccgtcatgg	cgttgaacctggctacttgg
*Col2a1*	gaaggtgctcaaggttctcg	ctccaggaataccatcagtcc
*Acan*	ggcagtggacttgtctcagg	ccttctccagcttcttgagc
*Mkx*	ttacaagcaccgtgacaacc	agccgacgtctagcattagc
*Col1a1*	gggcaagacagtcatcgaat	ggtggagggagtttacacga
*Col10a1*	caataggcagcagcattacg	gcgtgccgttcttatacagg
*Osx*	gaggttcactcgctctgacg	ctcaagtggtcgcttctgg
*Opn*	tctgatgagaccgtcactgc	cctcagtccataagccaagc
*Pax7*	gactccagctctgcctatgg	gagaggccattgctaacagg
*Pax3*	tcagcgagagagcatcagc	cagttgctctgctgtgaagg
*MyoD*	acggcatgatggagtacagc	tccgtgtagtagctgctgtcg
*Myosin*	aacctgcaacgtgtcaagc	cttctccagacttgccttgg
*Gapdh*	gggaagcccatcaccatct	cgacatactcagcaccggc
*Sca-1/Ly6a*	ggaggcagcagttattgtgg	gctacattgcagaggtcttcc
*Col14a1*	gccagtctgtctcaacacga	aatccttgcatgcctggtga
*Tnmd*	atgcagaagcatccaagacc	aagagcacgaggatgagagc
*Tgfi1*	acctgcctcctatccagagc	gtgcaacatccaccagtagc
*SnoN*	acctgcctcctatccagagc	ccacctcttgcagaatgagc

### Recombinant Limbs

Recombinant limbs were performed according to Ros et al. (1994). For each independent experiment, control, sham (GFP) and SCA1-electroporated cells were obtained from ∼30 freshly isolated 22HH chick hindlimb buds. After electroporation, cells were centrifuged at 1100 rpm and incubated at 37°C between 1.5 and 2 h to form a compact pellet. Separately, to obtain ectoderms, limb buds from ∼10 22HH embryos were dissected out in PBS, transferred into a tube, and digested in 0.5% trypsin in PBS for 30 min at 37°C. Incubated limb buds were transferred to PBS plus 10% FBS, and the ectoderm was peeled off. After incubation, the pellet of previously obtained mesodermal cells was detached from the bottom of the tube and transferred to a small petri dish containing the ectoderms. Later, a fragment of the pellet was stuffed into the ∼20 ectoderms. Immediately after the stuffed ectoderms were allowed to assemble for 15–20 min at room temperature, they were individually transferred into a previously windowed 22HH chick embryo and positioned between the somites 15–20 where a wound was previously scratched to attach it. Manipulated embryos were incubated for 48 h and 6 days at 38°C until they were collected (see Figure 4A). All RLs experiments were performed in triplicate. For each qRT-PCR independent replicate, RNA was obtained from a pool of twenty-five to thirty RLs for control, sham or SCA-1 condition. For each morphological experiment (Alcian blue and hematoxylin and eosin staining), at least ten embryos for each condition were manipulated. The frequency of the phenotypes is indicated in the text.

### Hematoxylin and Eosin Staining

Alcian blue-stained RLs were dehydrated with ethanol and xylol before being embedded in paraffin wax. Ten-micrometer sections were obtained with a Leica microtome RM2125 RTS and samples were rehydrated with an ascendant train of xylol–ethanol, then stained for 9 min with hematoxylin and 3 min with eosin dyes. Next, slides were dehydrated with ethanol–xylol and mounted with DPX medium (Sigma-Aldrich, cat. no. 44581). Images were acquired in a microscope, Olympus BX51-WI, equipped with fluorescence and a gyratory disk unit (Olympus Corporation, Tokyo, Japan) using the capture software Stereo Investigator v.9 (MicroBrightField Inc., Colchester, VT, United States).

### *In situ* Hybridization

RNA probes were labeled with UTP-digoxigenin (Roche Applied Science, Indianapolis, IN, United States) for their use in micromass *in situ* hybridization, as previously described by Chimal-Monroy et al. (2002). Samples were treated with 10 μg/mL of proteinase K (PK) for 5 min at 20°C for all genes. The hybridization and post-hybridization washes were at 65°C. Signal was visualized with a BM-Purple substrate for alkaline phosphatase (Roche Applied Science). Images were acquired in AxioZoom V.16 microscope (Carl Zeiss, Oberkochen, Germany) using Zen lite software (Carl Zeiss, Oberkochen, Germany).

### Immunofluorescence and Immunohistochemistry Assays

Mouse hindlimbs from CD-1 strain pregnant mice at E10.5 were fixed overnight in PFA 4% and dehydrated with sucrose. Samples were embedded in the Tissue-Tek OCT compound (Sakura, Torrance, CA, United States, cat. no. 4583) and frozen with dry ice. Twenty-micrometer slides were permeabilized with 0.3%-Triton X-100 in PBS and incubated with anti-CD29 (1:100, R&D Systems, Minneapolis, MN, United States, cat. no. AF2405), PE anti-mouse/human CD44 (1:300, Bio Legend, cat. no. 1030007), anti-CD73 (1:500, Abcam, Cambridge, MA, United States, cat. no. ab71822), anti-mouse Endoglin/CD105 (1:100, R&D Systems, cat. no. AF1320), and anti-mouse CD90/Thy1 (1:100, R&D Systems, cat. no. AF7335) antibodies at 4°C overnight. Samples were washed with PBS 1× and incubated for 2 h at room temperature with secondary antibodies, donkey anti-rat biotinylated (Merck Millipore, cat. no. AP189B), Alexa Fluor^®^ 488 donkey anti-goat (cat. no. A11055), Alexa Fluor^®^ 555 donkey anti-sheep (cat. no. A21436), Alexa Fluor^®^ 647 donkey anti-rabbit (cat. no. A31573), or Alexa Fluor^®^ 488 donkey anti-rat (cat. no. A21208), all acquired from Molecular Probes. To reduce the autofluorescence in the samples, they were incubated for 45 min at room temperature with CuSO_4_ 1 mM in NH_4_ 50 mM pH 5 before the addition of the primary antibody. Nuclei were stained with DAPI (1 mg/ml, Sigma-Aldrich). Samples were mounted with Dako Fluorescence Mounting Medium (Agilent, Santa Clara, CA, United States). The images were acquired with a vertical microscope, Olympus BX51-WI, coupled with a spinning disk unit (Olympus Corporation) using the program Stereo Investigator v.9 (MicroBrightField Inc., Colchester, VT, United States). For SCA-1 immunohistochemistry detection (1:300, R&D Systems cat. no. BAM1226), the same general protocol was followed in addition to 3% H_2_O_2_ incubation before triton 100× permeabilization. After secondary antibody addition, samples were incubated with the VectaStain ABC Kit (Vector Laboratories, Burlingame, CA, United States, cat. no. PK-6100), and positive cells were visualized with the DAB Peroxidase Substrate Kit (Vector Laboratories, cat. no. SK-4100). Immunohistochemistry images were acquired in AxioZoom V.16 microscope (Carl Zeiss, Oberkochen, Germany) using Zen lite software (Carl Zeiss, Oberkochen, Germany).

### Alcian Blue Staining

For RLs, samples were fixed in 5% trichloroacetic acid (Sigma-Aldrich) for 24 h and stained with 1% Alcian blue in ethanol-HCl for 24 h. Following staining, RLs were transferred to 100% ethanol for 24 h and cleared with methyl salicylate (Sigma-Aldrich) until the skeleton was observed. Images for RLs were acquired with the SMZ1500 microscope (Nikon, Tokyo, Japan).

For micromass cultures, cells were incubated in Khale’s fixative for 20 min and stained with 0.5% Alcian blue for 24 h, then washed twice with HCl 0.1 N and stored at 4°C in 2% PFA in PBS. Images for micromass cultures were acquired in the AxioZoom V.16 microscope (Carl Zeiss, Oberkochen, Germany) using Zen lite software (Carl Zeiss, Oberkochen, Germany). Nodules quantitation represents three independent experiments.

## Results

### The Localization of the Primary MSC Markers Is Not Restricted to Mesodermal Cells in the Early Limb Bud

**FIGURE 1:**
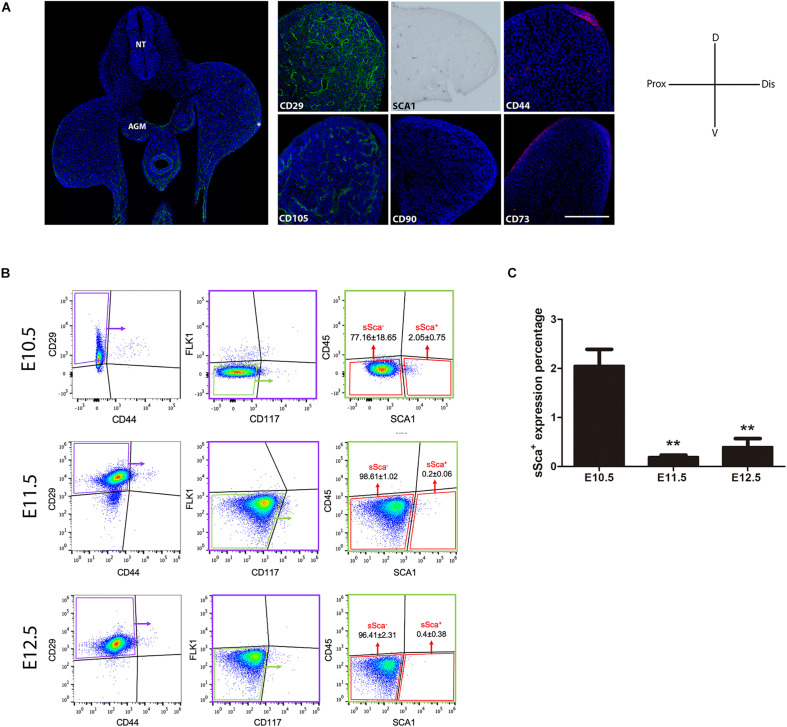
*In vivo* detection of two MSP limb bud subpopulations based on MSC-am localization. **(A)** Transversal section of an E10.5 mouse embryo to show the histological organization at this stage; the immunodetections of CD29, SCA-1, CD44, CD105, CD90, and CD73 markers in E10.5 limb buds are shown. The limb bud for each MSC-am marker corresponds to the region inside the square in panel **(A)**. **(B)** Dot plots of the multiparametric flow cytometry analysis showing the hierarchy to identify sSca^+^ and sSca^–^ subpopulations in E10.5, E11.5, and E12.5 mouse limbs. The number of positive cells for each subpopulation is expressed in percentage; ±mean standard deviation. **(C)** Comparison of the expression percentage of the sSca^+^ subpopulation between E10.5, E11.5, and E12.5 limbs. Statistical significance was set as follows: E10.5 vs. E11.5 *p* > 0.0061; 10.5 vs. 12.5 *p* > 0.0023; E11.5 vs. E12.5 *p* > 0.4079, **p < 0.005. NT, neural tube; AGM, aorta-gonad-mesonephros; Do, dorsal; V, ventral; Pr, proximal; D, distal. In **(A)** AER is marked with an asterisk. The scale bar is set at 200 μm and is representative for all images.

We analyzed the expression pattern of the mainly MSC markers CD29, Sca-1, CD44, CD105, CD90, and CD73 in E10.5 hindlimb buds. Our analysis revealed that only mesodermal tissue is positive for CD29 and Sca-1, whereas CD44 and CD105 were in the apical ectodermal ridge (AER) and endothelial cells, respectively. Also, only dorsal ectoderm cells were positive for CD73. Finally, we did not detect positive cells to CD90 in E10.5 limb buds (Figure 1A). These results demonstrated that despite the expression of a variety of MSC-am during limb development, not MSC markers are exclusively expressed in mesodermal cells *in vivo*.

### Two Well-Defined Mesodermal Skeletal Progenitor Subpopulations Based on SCA-1/Ly6A Expression Can Be Distinguished in Limb Buds

Taking into consideration the expression pattern of the MSC-am in limb cells, we performed a multiparametric FACS analysis to identify MSP subpopulations exclusively in the early E10.5 limb bud. SCA-1 is a known hematopoietic stem cell marker (Spangrude et al., 1988); consequently, in addition to MSC markers, we considered a negative selection for CD117, CD45, and FlK1 to rule out the possibility of isolating hematopoietic and endothelial cells. Thus, the combination of CD29, SCA-1, CD44, Flk1 (instead of CD105, see Supplementary Figure 1), CD45, and CD117 markers allowed us to identify, in freshly isolated limb bud cells, two different subpopulations: the Sca-1-negative subpopulation herein called sSca^–^ (CD29^+^, CD44^–^, Flk1^–^, CD45^–^, CD117^–^, and SCA-1^–^), which corresponded to the 77.16 ± 18.65%, and the Sca-1-positive subpopulation herein called sSca^+^ (CD29^+^, CD44^–^, Flk1^–^, CD45^–^, CD117^–^, and SCA-1^+^), which corresponded to the 2.05 ± 0.75% of total E10.5 hindlimb cells (Figure 1B). To investigate the cellular dynamics of both subpopulations during development, we evaluated the percentage for sSca^+^ and sSca^–^ subpopulations at 11.5 and E12.5 hindlimb. In E11.5 hindlimbs, subpopulations comprised 0.2 ± 0.06% and 98.61 ± 1.02% for sSca^+^ and sSca^–^, respectively. On the other hand, in E12.5 hindlimb cells, sSca^+^ and sSca^–^ subpopulations encompassed 0.4 ± 0.38% and 96.41 ± 2.31%, respectively (Figure 1B). These results revealed that the sSca^+^ subpopulation significantly diminished through development while the sSca^–^ subpopulation was maintained with no significant change (Figure 1C), suggesting that the sSca^+^ subpopulation diminished concomitantly with limb development.

### sSca^+^ and sSca^–^ Subpopulations Possess Dissimilar Gene Expression Profiles and *in vitro* Differentiation Capacity Into Limb Lineages

Considering that most, if not all, cells in the E10.5 stage limb bud are histologically indistinguishable and, in comparison to the E11.5 and E12.5 stages, the amount of the sSca^+^ subpopulation is higher, we decided to characterize both sSca^+^ and sSca^–^ subpopulations in the E10.5 stage without previous culture expansion. Immediately after their isolation, we analyzed by qRT-PCR the differences in *Sca-1* expression between subpopulations confirming that sSca^+^ express almost two-fold more *Sca-1* than sSca^–^ cells (*p* > 0.0449; Supplementary Figure 2).

**FIGURE 2:**
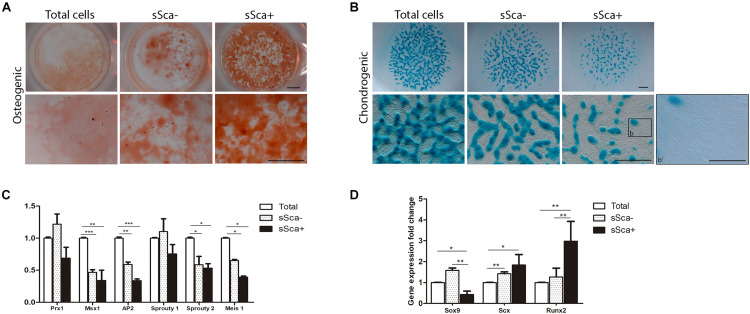
sSca^+^ and sSca^–^ limb bud subpopulations are differentially committed to limb lineages. **(A)** *In vitro* osteogenic (alizarin red staining) and **(B)** chondrogenic (Alcian blue staining) differentiation capacity of freshly isolated sSca^+^ and sSca^–^ subpopulations compared to total cells. Inset (b) corresponds to magnification showed in b′. Comparison of the expression profile by qRT-PCR of undifferentiated zone genes **(C)** and master differentiation genes **(D)** between sSca^+^, sSca^–^ subpopulations and total cells. Data represent three independent experiments. Statistical significance was set as follows: ***p < 0.0001, **p < 0.005, *p < 0.05. Scale bar set is at 1 mm for all images in panels **(A,B)**, for b′ is set at 100 μm.

Furthermore, to determine the commitment stage of both sSca^–^ and sSca^+^, freshly sorted subpopulations were cultured under specific differentiation conditions for osteogenic and chondrogenic lineages and compared with total cells. The results showed that both subpopulations had a remarkably osteogenic differentiation capacity in comparison to total cells, although the sSca^+^ cells presented higher osteogenic differentiation than the sSca^–^ cells (Figure 2A). In contrast, the sSca^–^ subpopulation and total cells seeded in micromass cultures showed a higher chondrogenic differentiation capacity than sSca^+^ cells. Remarkably, in the sSca^+^ micromass cultures, abundant cells with fibroblastoid morphology were observed adjacent to cartilage nodules (Figure 2B). Notably, fibroblastoid cells appear as individual cells with no apparent cell fusion, suggesting the possibility that they might not represent muscle cells (Figure 2B, arrowheads).

To confirm whether both subpopulations were intrinsically distinct from each other and from total cells, we evaluated, immediately after its isolation by FACS, the expression profile of genes expressed in limb-undifferentiated cells (Tabin and Wolpert, 2007) and master genes for skeletal tissues. qRT-PCR analysis from sSca^–^, sSca^+^ subpopulations and total freshly isolated cells showed no statistically significant changes in the expression of limb undifferentiated related genes between both subpopulations. However, the expression of *Msx1*, *AP2*, *Sprouty 2*, and *Meis1* was lower in both subpopulations than total cells (Figure 2C). In contrast, the evaluation of master differentiation genes showed a reduction in the expression of *Sox9* in the sSca^+^ subpopulation in comparison to sSca^–^ (*p* < 0.002) and with total cells (*p* < 0.046). Also, the levels of *Runx2* expression were higher in the sSca^+^ than sSca^–^ (*p* < 0.002) and total cells (*p* < 0.0086), while there was no significant change in *Scx* (*p* < 0.12) expression between both subpopulations (Figure 2D). However, *Scx* expression is higher in sSca^–^ (*p* < 0.0034) and sSca^+^ (*p* < 0.034) than total cells. Because we observed a high osteogenic capacity in both subpopulations and the formation of tendon-like cells in sSca^+^, we decided to evaluate the expression of *Col2a1*, *Aggrecan*, *Mohawk*, *Col1a1*, *Col10a1*, *Osterix*, and *Osteopontin* in both subpopulations. We found no significant statistical changes in the expression of these genes between subpopulations (data not shown). Taken together, the qRT-PCR analysis and cell differentiation experiments showed that the expression of SCA-1 in combination with a positive and negative selection of MSC-am allows to distinguish of two independent MSP subpopulations in the limb bud with a specific commitment stage.

### *Sca-1* Expression in Limb Bud Mesodermal Cells Induces Tenogenic Commitment

**FIGURE 3:**
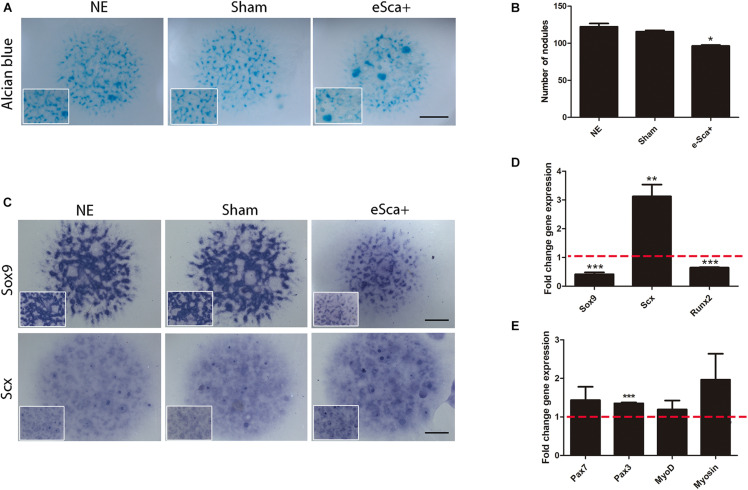
*Sca-1* expression is sufficient to induce *Scx* and inhibits chondrogenesis. Three days of micromass cultures of non-electroporated (NE), sham, and Sca-1 electroporated limb bud cells (eSca-1^+^). **(A)** Chondrogenic phenotype by Alcian blue staining and **(B)** Nodule quantitation are shown. **(C)** *In situ* hybridization of *Sox9* and *Scx*. Expression profile by qRT-PCR of master differentiation genes of chondrogenic, tenogenic, and osteogenic lineages **(D)** and myogenic genes **(E)** in eSca-1^+^ cells. Expression relative to sham cells is shown (set to 1.0, dashed red line). Data represent at least three independent experiments. Statistical significance represents ***p < 0.0005, **p < 0.005, *p < 0.05. Scale bar is set at 2 mm and is representative for all images.

The next step was to evaluate whether *Sca-1* expression was sufficient to induce cellular commitment of specific skeletal lineages. We used limb bud chick mesodermal cells, which lacks Sca1/Ly6a gene, instead of mouse limb cells to avoid interference in the results for the acquisition of SCA-1 under culture conditions (Marín-Llera and Chimal-Monroy, 2018). The Sca-1-plasmid was electroporated in mesodermal cells from 22HH chick hindlimb buds, the equivalent developmental stage of E10.5 in the mouse embryo. Measurements by flow cytometry showed that after a 3-day culture a 64.4 ± 0.6% of SCA-1-transfected cells were still positive for this marker (Supplementary Figure 3). Under the same conditions, we explored gene regulation by *Sca-1* in 3-day micromass cultures because, at this time, chondrogenic differentiation is evident. Alcian blue staining of *Sca*-electroporated cells, herein called e-Sca^+^, also showed a reduction in their chondrogenic potential (Figures 3A,B), as was observed with the recently isolated sorted sSca^+^ subpopulation (compare with Figure 2A). The detected downregulation of *Sox9* confirmed this observation. Further, we observed the upregulation of *Scx* in e-Sca^+^ cells (Figure 3C). Transcriptional level characterization of the micromass cultures of e-Sca^+^ cells showed that *Sca-1* upregulates *Scx* (*p* < 0.0065) while the expression of *Sox9* (*p* < 0.0001) and *Runx2* (*p* < 0.0001) decreased (Figure 3D). Considering the morphology of elongated cells in the sSca^+^ observed after high-density cultures (see Figure 2A), and that an SCA-1 subpopulation was previously reported only as myogenic progenitors (Reinhardt et al., 2019), we evaluated whether *Sca-1* promoted myogenic differentiation. The results showed that in 3-day micromass cultures, there was a significant change in the expression of *Pax3* (*p* < 0.0001) but *Pax7* (*p* < 0.20), *MyoD* (*p* < 0.13), and myosin (*p* < 0.15) genes remained without significant changes (Figure 3E). Together, the results showed that *Sca-1* expression diminishes chondrogenic differentiation through the downregulation of *Sox9*, favoring the tenogenic differentiation of limb MSP cells, and myogenesis was not induced.

### Morphogenetic and *in vivo* Differentiation Capacity of Mesodermal SCA-Positive Cells

**FIGURE 4:**
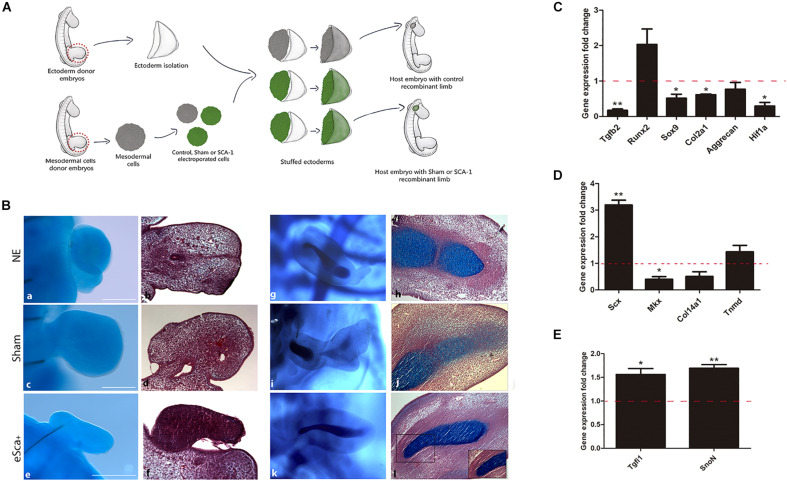
The morphogenetic capacity of *Sca-1* limb bud cells in the recombinant limb model. **(A)** Schematic representation of the experimental design of recombinant limbs experiments. Recombinant limbs (RLs) were performed with non-electroporated (NE), sham (GFP), and *Sca-1* electroporated limb bud cells (e-Sca-1^+^). Mesodermal hindlimb cells and ectoderms were obtained from 22HH embryos. Electroporated (GFP or e-Sca-1) and NE recombinant limbs were implanted in 22HH chick embryos and collected after 2 **(a–f)** or 6 **(g–l)** days. **(B)** Alcian blue stain to evidence skeletal elements **(a,c,e,g,i,k)**. Sagittal slices of RL after Alcian blue staining stained with hematoxylin and eosin **(b,d,f,h,j,l)**. Square delimits the magnification for b′,d′,f′. Scale bars are representative for all conditions representing 100 μm. qRT-PCR of chondrogenic **(C)**, tenogenic **(D)**, and tendon cell fate regulating genes **(E)** of the e-Sca^+^ 48h RLs relative to the GFP-RLs is shown (set to 1.0, dashed red line). GFP-RLs and NE-RLs showed no statistical differences between all genes (data no show). Data represent three independent experiments. Statistical significance was set as follows: **p < 0.005, *p < 0.05.

Gain-of-function assays showed that *Sca-1* is important to regulating the tenogenic, osteogenic, and chondrogenic genes of limb cells *in vitro*. However, to determine the role of *Sca-1* in morphogenesis and differentiation *in vivo*, we used as an experimental model the generation of RLs with non-cultured e-Sca^+^ cells. First, as a control to ensure that the RLs model is suitable for evaluating electroporated cells, we used GFP-electroporated chick mesodermal hindlimb cells from the 22HH stage to generate RLs. The results showed that GFP-Recombinant Limbs (GFP-RL) were developed with GFP-cells (Supplementary Figure 4). This suggested that cell electroporation did not interfere with RLs formation. Therefore, we performed RLs with control- (non-electroporated), sham- (electroporated with GFP plasmid), and e-Sca-1^+^ cells (Figure 4A). The results revealed a lack of central skeletal elements with non-electroporated (NE; 5/5), sham (6/6), and e-Sca-1^+^ (8/8) cells after 48 h of development (Figures 4Ba–f). This time allows elucidating the role of SCA-1 at the early commitment of mesodermal cells. However, the histological analyses showed central condensations in control and sham conditions, while in e-Sca-1^+^ RL are differentially organized (Figure 4Bb,d,f). Remarkably, after 6 days, RLs revealed a formation of a central and segmented skeletal element in control (2/2), sham (2/2), and e-Sca^+^ (1/2). Moreover, a high proportion of elongated cells is maintained in e-Sca^+^ RL (2/2) (Figure 4Bg–l). To distinguish the early commitment of mesodermal cells exhibited by the SCA-1 expression, we did a molecular characterization of chondrogenic, osteogenic, and tenogenic genes in 48 h RLs (Figures 4C–E). Expression analysis demonstrated that *Tgfb2* (*p* < 0.0048), *Sox9* (*p* < 0.046), *Col2a1* (*p* < 0.013), and *Hif1a1* (*p* < 0.033) expression was significative diminished, while aggrecan (*p* < 0.328) and *Runx2* (*p* < 0.15) expression remains without significative changes in e-Sca^+^ RLs in comparison with sham RLs (Figure 4C). Remarkably, *Scx* (*p* < 0.007) expression is upregulated while *Mkx* (*p* < 0.043) is diminished. *Col14a1* and *Tnmd*, genes expressed in more advanced stages of tenogenesis, remain without significant changes (*p* < 0.125 and *p* < 0.52, respectively). However, a clear tendency of *Tnmd* upregulation is observed (Figure 4D). Besides, we evaluated the expression of *Tgfi1* and *SnoN* because these proteins negatively regulate TGFβ signaling and are involved in the induction of common precursor cells to enter the tendon differentiation program instead of chondrogenesis (Lorda-Diez et al., 2009). Our results revealed that in the presence of SCA-1, *Tgfi1* (*p* < 0.049), and *SnoN* (*p* < 0.0048) are upregulated in 48 h RLs (Figure 4E). These data support the idea that the *Sca-1* expression in limb mesodermal cells affects chondrogenesis *in vivo*, promoting tenogenic commitment by inhibiting TGFβ signaling through *Tgfi1* and *SnoN*.

## Discussion

During development, inductive signals control the temporal and spatial patterns of tissue differentiation (reviewed by Tabin and Wolpert, 2007; Marín-Llera et al., 2019). However, the question of whether a homogenous population of multipotent progenitors forms the early limb bud or whether progenitors become precociously committed to specific fates remains to be clarified. In the adult mouse limb, different skeletal progenitor cell subpopulations were identified: the mSSC (Chan et al., 2015), the PαCD51 (Pinho et al., 2013), and the PαS (Morikawa et al., 2009). The embryonic origin of the progenitors was traced up to advanced stages of development (Nusspaumer et al., 2017). In a more recent study, Reinhardt et al. (2019) identified three distinct limb cell populations in the posterior distal mesoderm and the core and peripheral mesoderm of mouse forelimbs at E10.5–E10. In agreement with these observations, our study supports the idea that the mesoderm of early mouse limb buds is already composed of different types of progenitors with different specification and differentiation states.

In the present work, isolation of the sSca subpopulations was based on a panel of MSC markers. In their study, Reinhardt et al. (2019) identified two distinct cell populations based on the expression of SOX9, PDGFRα, SCA, JAG, and Lin. SCA^–^ subpopulation constituted 25% of forelimb cells, whereas SCA-1^+^ corresponded to 6%. In both cases, they represent three times the sSca cells identified in the present work, supporting that sSca cells identified here might represent different subpopulations to that established by Reinhardt et al. (2019). Interestingly, unlike Reinhardt et al. (2019), our qRT-PCR analysis demonstrated that the total and sSca^–^ cells presented higher levels of *Sox9* than sSca^+^ cells. In this sense, the limb cellular subpopulations isolated based on the SCA-1 marker may have a different commitment stage, and SCA^+^ cells are not necessarily restricted to muscle lineage as previously reported (Reinhardt et al., 2019).

The two subpopulations of MSP cells (sSca^+^ and sSca^–^), identified here in the early limb bud, did not express *Pprg* and *Adiponectin* genes (data not shown), supporting their commitment to skeletal lineages. However, both subpopulations follow distinct fates during further development. sSca^+^ subpopulation diminished throughout development while the sSca^–^ subpopulation did not change. Additionally, there was no difference between both populations in the expression of genes related to undifferentiated cells, but some of these genes have lower expression in both subpopulations compared to total cells. When Sca^+^ subpopulations are directly isolated from loose mesenchyme cells of E10.5 limb buds, they showed higher expression of *Runx2*, lower *Sox9* and no changes in *Scx* expression compared to total and sSca^–^. In concordance with this expression profile, sSca^+^ cells seeded in monolayer culture conditions showed higher osteogenic differentiation capacity than in sSca^–^ and total cells. Interestingly, in total cell conditions, the final osteogenic cell density appears lower than in both subpopulations. However, this lower osteogenic potential does not allow discarding that a number of these osteoprogenitors die during the 15-days of culture. In support of the different commitment stage of the Sca-subpopulations identified here, elongated cells were predominantly found in the high-density culture of sSca^+^ cells but not in monolayer cultures. At the same time, chondrogenesis predominated in the culture of total and sSca^–^ cells. This observation led us to hypothesize that cell density could be necessary to cell fate decisions in *Sca-1* expressing cells, supporting that sSca^+^ cells differentiate into dense connective tissue and eventually tendon or bone tissue. Interestingly, neither the sSca^–^ nor sSca^+^ cells have more chondrogenic capacity than total cells. These findings are summarized in Table 2. Taking together, the expression profile of both subpopulations identified here, and its behavior under differentiation inductive signals, suggests that both may represent early committed cells. Therefore, limb bud mesodermal cells may be a heterogeneous population that contains several osteoprogenitors.

**TABLE 2:** Behavior of mouse SCA-1-subpopulations and chicken SCA-1-electroporated cells based on its phenotype and gene expression profile of master differentiation genes.

	Gene expression in freshly isolated cells	*In vitro* differentiation			*In vivo* differentiation (Recombinant limbs)	
			
	Master genes	Monolayer	High density micromass culture		Patterning signals from limb ectoderm	
				
		Osteogenesis	Chondrogenesis/osteogenesis	Tenogenesis	Chondrogenesis/osteogenesis	Tenogenesis
**sSca^+^** (mouse)	*Runx2* (2.98 ± 0.94) *Sox9* (0.43 ± 0.16) *Scx* (1.84 ± 0.50)	High (+++)	Low (+)	High (+++)	ND	ND
**sSca^–^** (mouse)	*Runx2* (1.26 ± 0.42) *Sox9* (1.57 ± 0.11) *Scx* (1.42 ± 0.08)	High (+++)	Medium (++)	Low (+)	ND	ND
**Total limb bud cells** (mouse)	*Runx2*, *Sox9*, and *Scx* (1.0)	Low (+)	High (+++)	Low (+)	ND	ND
**e-Sca^+^** (chicken)	ND	ND	Low (+) *Sox9* (0.38 ± 0.12) *Runx2* (0.64 ± 0.02)	High (+++) *Scx* (2.93 ± 0.45)	Low (+) *Sox9* (0.51 ± 0.04) High (+++) *Runx2* (2.03 ± 0.15)	High (+++) *Scx* (3.22 ± 0.007)
**e-Sham** (chicken)	ND	ND	High (+++) *Sox9* and *Runx2* (1.0)	Low (+) *Scx* (1.0)	High (+++) *Sox9* and *Runx2* (1.0)	Low (+) *Scx* (1.0)

*High, medium, and low represent the observed phenotype for each condition. Total cells (mouse) or e-Sham (chicken) were set to 1.0 to calculate gene expression fold change. All gene expression data were taken from Figures 2–4 and are presented as mean ± SD. ND = No determined.*

The next step in this study was to evaluate the role of SCA-1 expression in mouse limb cells. However, after *in vitro* culture, SCA-1 expression is acquired in most cells derived from mouse embryonic limbs and other tissues (Marín-Llera and Chimal-Monroy, 2018). For this reason, we decided to evaluate in chick limb bud mesodermal progenitors, which lacks Sca1/Ly6a gene, the role of SCA-1 expression to induce cellular commitment. For this, we used as *in vitro* approach micromass cultures, and as in *in vivo* model, RLs. The gain-of-function strategy demonstrated that under high-density culture conditions, *Scx* was highly expressed while *Sox9* is diminished, suggesting that SCA-1 is sufficient to regulate the earliest steps of the tenogenic lineage positively. Besides, using the RLs model as an *in vivo*-like environment where e-Sca-1 cells were under the influence of limb patterning signals from limb ectoderm, SCA-1 was sufficient to regulate *Scx* and *Runx2* positively and negatively *Sox9*. On this basis, we suggest that tenogenic and osteogenic lineages are promoted instead of chondrogenic under the influence of Sca-1. Remarkably, in RLs at day 2, the levels of *Sox9* in e-Sca^+^-RLs were lower than RLs with sham cells, suggesting that cartilage lineage is less favored in these conditions (see Table 2). Interestingly, after 6 days, skeletal elements were evident in e-Sca^+^-RLs, it might indicate that commitment of e-Sca-1 cells to cartilage lineage is posterior to tenogenic or osteogenic lineage. Thus, because *Scx* expression is higher than *Sox9* at day 2 in RLs, the densely packed cells observed might correspond to cellular aggregations committed to tenogenic linage as it has been described by Ros et al. (1995). The lower expression of *Sox9* and *Hif1a* together with *Col2A* supports this idea.

During limb development, chondrocytes and tenocytes differentiate from a common precursor expressing *Sox9* and *Scx*. Precursor cells become Scx^+^/Sox9^–^ when received signals that promote tendon differentiation. In contrast, cells become Scx^–^/Sox9^+^ once chondrocyte differentiation is promoted (Blitz et al., 2013; Sugimoto et al., 2013). In this study, in the RLs at day 2, we observed up-regulation of *Scx*, whereas the *Mkx* gene was downregulated. It is known that *Mkx* is gradually expressed during development, thus here is probable that *Mkx* expression is starting at the evaluated time point. *Scx* expression can explain the increase of *Tnmd* because it positively regulates the expression of *Tnmd*, and it does in a tendon lineage-dependent manner (Shukunami et al., 2006). Interestingly, *Mkx* promotes *Scx* expression through binding to the *Tgfb2* promoter (Liu et al., 2015). Thus, because *Tgf*β2 is downregulated in RLs, the initial *Scx* induction we observed may be TGFβ-independent (Pryce et al., 2007; García-Lee et al., 2021). On the other hand, we also found upregulation of *Tgfi1* and *SnoN*. It is known that both gene products participate in inhibiting TGFβ signaling (Lorda-Diez et al., 2009). In this context, it is reasonable to speculate that SCA-1 expression may participate, allowing that committed cells differentially respond to the threshold of the TGFβ signaling promoting to enter the tenogenic or chondrogenic differentiation program.

On the other hand, we observed that the SCA-1 expression is also important for cell commitment of osteoblastic lineage. In other studies, it was determined that after an injury, SCA1-positive cells from the Achilles tendon give rise to ectopic bone formation (Agarwal et al., 2017). Moreover, SCA1-expressing tendon cells can differentiate into osteocytes, in contrast to peritendon cells that fail to differentiate into this lineage (Mienaltowski et al., 2013). On this basis, the expression of *Runx2* in Sca-1^+^ cells may be necessary to promote endochondral ossification in later stages of development. In this context, we suggest that the sSca^+^ subpopulation might be maintained at lower levels during development and adulthood, playing a role in osteo- and tenogenic differentiation in adults.

Whether SCA-1 regulates cell proliferation or cell death in the limb remains unknown. However, studies in muscle cell lines suggest that SCA-1 expression inhibits proliferation while differentiation is promoted (Epting et al., 2004; Mitchell et al., 2005). Interestingly, the size of micromass cultures and RLs with e-Sca^+^ cells appear to be reduced compared to non-electroporated and sham cells. This observation could be related to an effect in cell proliferation of SCA-1 expression in limb mesodermal cells.

In conclusion, our work provides evidence of the possibility of recognizing progenitor subpopulations in the limb according to the pattern expression of MSC markers. Further, it supports the idea that the limb bud mesodermal cells represent different progenitors with differential commitment states and different responsiveness to inductive signals from the early stages of development. We propose that the sSca-1^+^ subpopulation corresponds to early committed progenitors, giving rise to tenogenic and osteogenic lineages in the limb. Depending on the cellular context, *Sca-1* expression drives the cell fate for these lineages, probably acting as a permissive factor, while the ability to differentiate into chondrogenic capacity diminishes.

## Data Availability Statement

The raw data supporting the conclusions of this article will be made available by the authors, without undue reservation.

## Ethics Statement

The animal study was reviewed and approved by Committee for the Care and Use of Laboratory Animals Institute of Biomedical Research, National Autonomous University of Mexico.

## Author Contributions

JM-L, JH, and JC-M conceived and designed the experiments. JM-L and CL-D performed the experiments. JM-L and JC-M wrote the manuscript. All authors analyzed the data, revised and approved the final manuscript.

## Conflict of Interest

The authors declare that the research was conducted in the absence of any commercial or financial relationships that could be construed as a potential conflict of interest.

## Supplementary Material

Supplementary Figure 1 Co-expression of CD105 and FLK1 in E10.5 hindlimbs. Transversal section of an embryo at the E10.5 stage. Here co-localization by immunofluorescence of CD105 and FLK1 proteins is evident. Inset represents a magnification of the dashed line zone. Interestingly, there are some CD105^+^ FLK1^–^ cells, mostly localized in the periphery of the E10.5 hindlimb buds. Nuclei are stained with DAPI. Scale bars represent 100 μm in both images.

Supplementary Figure 2 qRT-PCR analysis of *Sca-1* expression in recently isolated sSca^–^ and sSca^+^ subpopulations. Expression relative to sSca^–^ cells is shown (set to 1.0, dashed red line).

Supplementary Figure 3 Electroporation efficiency of *Sca-1* in limb bud cells. Histograms of the flow cytometry determination of SCA-1-positive cells after electroporation with *Sca-1*-plasmid are shown. **(A)** Unstained electroporated cells after 3 days in the micromass culture. **(B)** Electroporated cells stained with anti-SCA-1-PE/Cy7 after 3 days in the micromass culture. The main population was determined by forward and side scatter, and only single cells were analyzed. Data are expressed in percentage and represent two independent experiments; ± mean standard deviation.

Supplementary Figure 4 Recombinant limb formation with GFP electroporated limb bud cells. Thirty-hour recombinant limb performed with GFP electroporated 22HH hindlimb bud cells. Data represent three independent experiments. Scale bar: 100 μm.
