# Supplementary figures and images for: SCA-1/Ly6A Mesodermal Skeletal Progenitor Subpopulations Reveal Differential Commitment of Early Limb Bud Cells
Source: Front Cell Dev Biol. 2021 Jul 16;9:656999. doi: 10.3389/fcell.2021.656999 (PMC8322737; doi:10.3389/fcell.2021.656999)

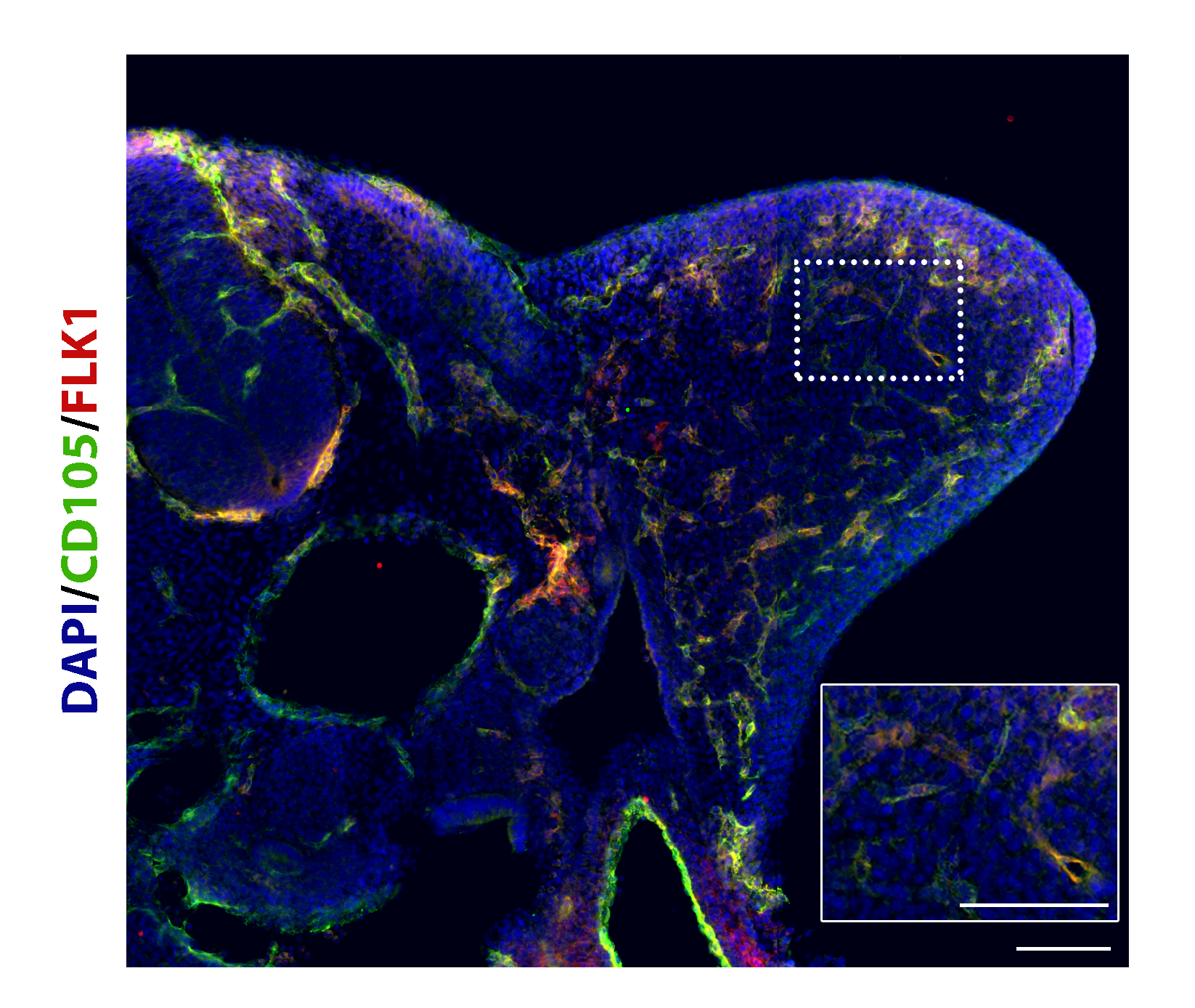

Supplement: Supplementary Figure 1 — Co-expression of CD105 and FLK1 in E10.5 hindlimbs. Transversal section of an embryo at the E10.5 stage. Here co-localization by immunofluorescence of CD105 and FLK1 proteins is evident. Inset represents a magnification of the dashed line zone. Interestingly, there are some CD105+ FLK1– cells, mostly localized in the periphery of the E10.5 hindlimb buds. Nuclei are stained with DAPI. Scale bars represent 100 μm in both images. [file Image_1.TIF]

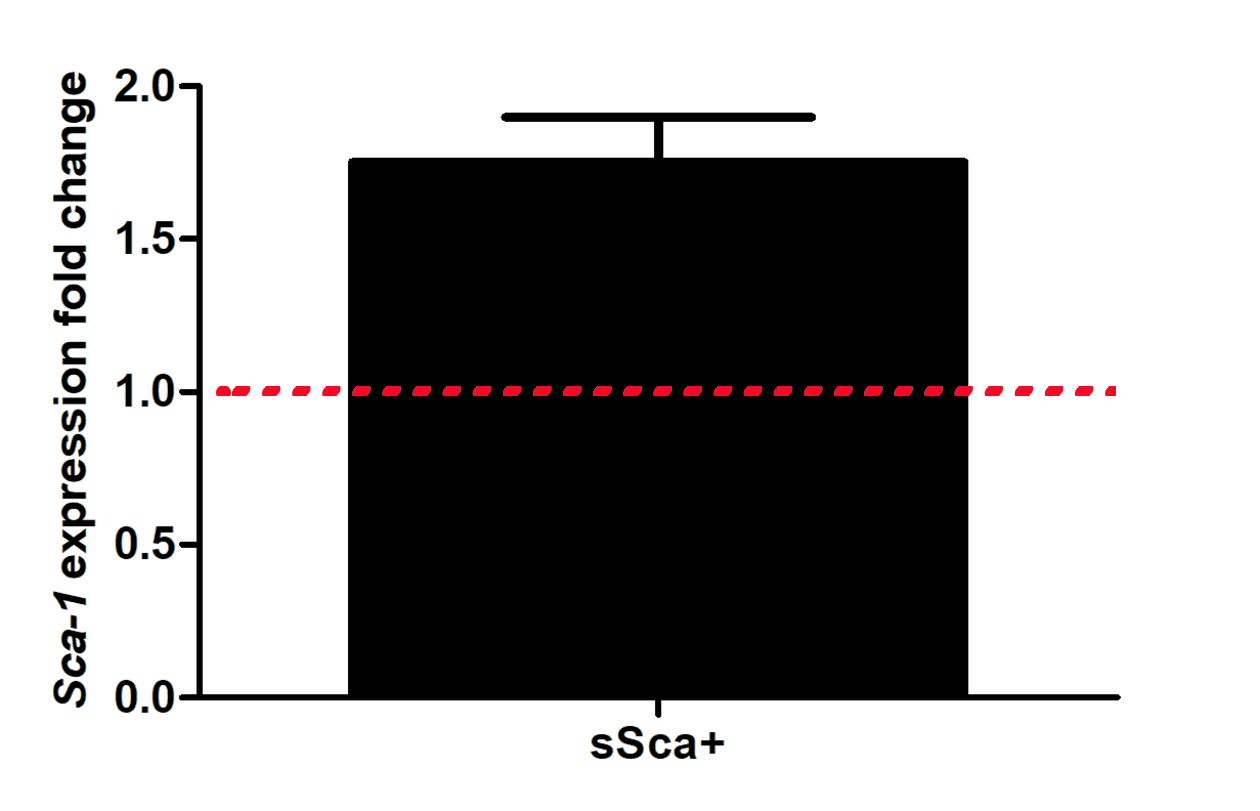

Supplement: Supplementary Figure 2 — qRT-PCR analysis of Sca-1 expression in recently isolated sSca– and sSca+ subpopulations. Expression relative to sSca– cells is shown (set to 1.0, dashed red line). [file Image_2.TIF]

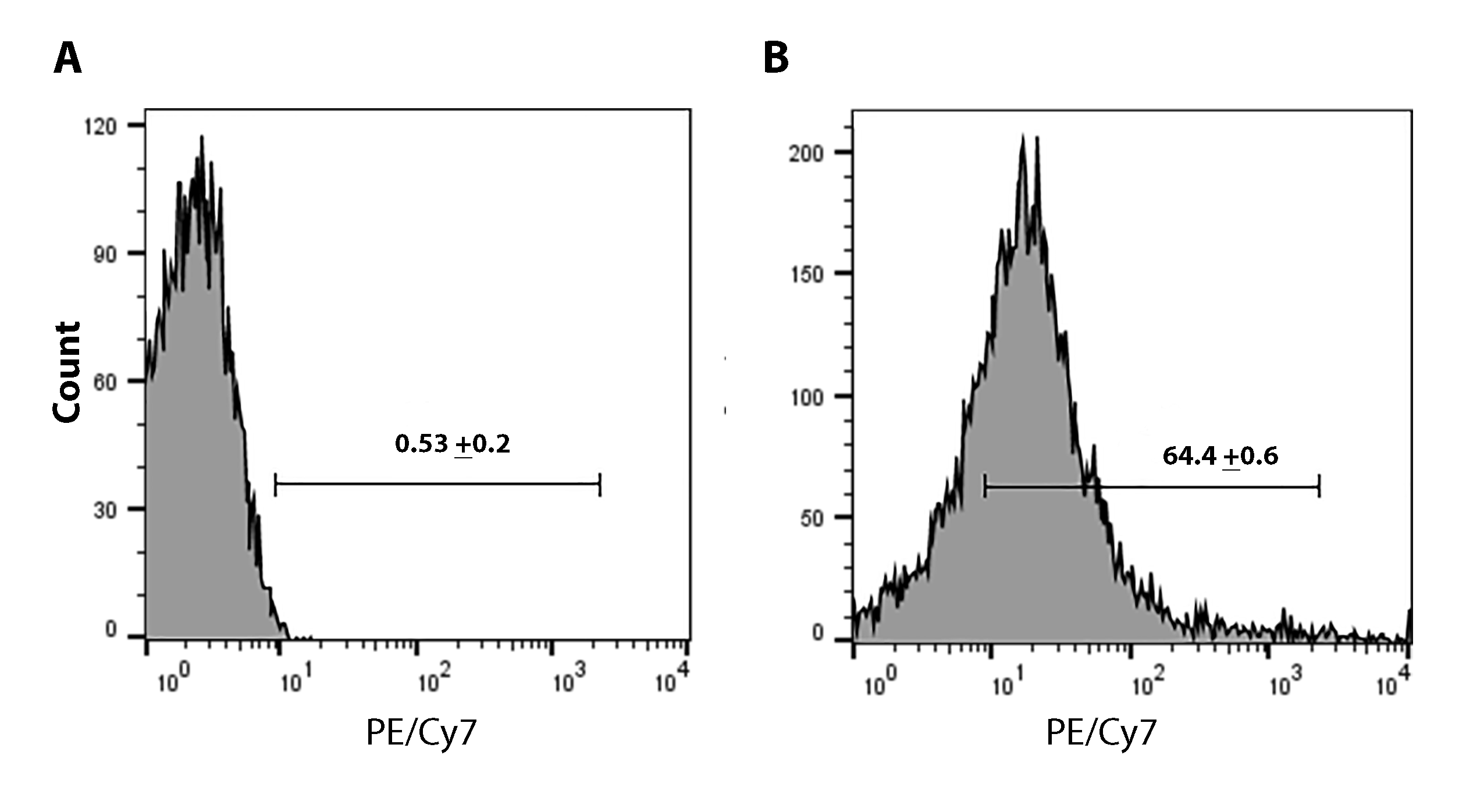

Supplement: Supplementary Figure 3 — Electroporation efficiency of Sca-1 in limb bud cells. Histograms of the flow cytometry determination of SCA-1-positive cells after electroporation with Sca-1-plasmid are shown. (A) Unstained electroporated cells after 3 days in the micromass culture. (B) Electroporated cells stained with anti-SCA-1-PE/Cy7 after 3 days in the micromass culture. The main population was determined by forward and side scatter, and only single cells were analyzed. Data are expressed in percentage and represent two independent experiments; ± mean standard deviation. [file Image_3.TIF]

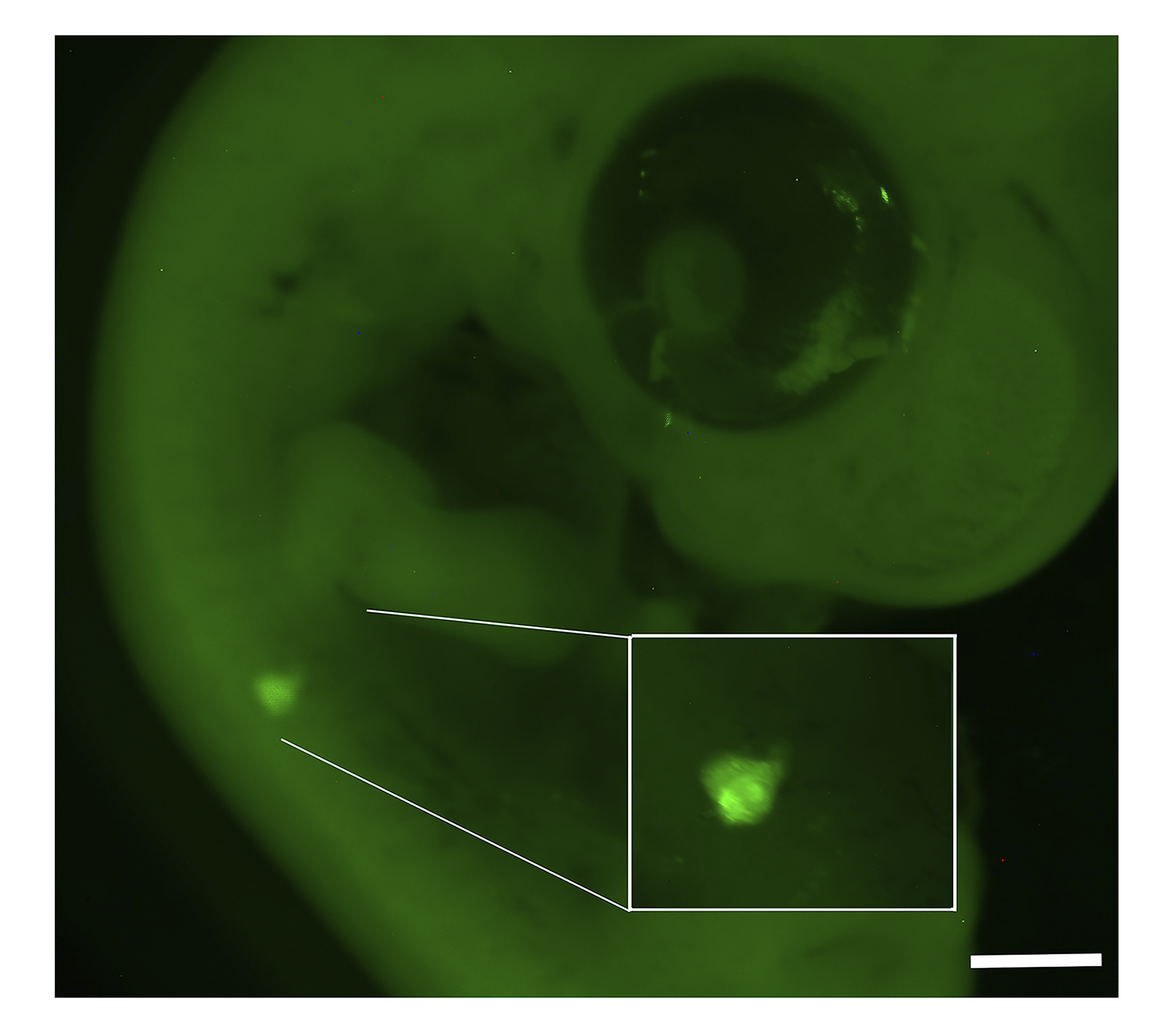

Supplement: Supplementary Figure 4 — Recombinant limb formation with GFP electroporated limb bud cells. Thirty-hour recombinant limb performed with GFP electroporated 22HH hindlimb bud cells. Data represent three independent experiments. Scale bar: 100 μm. [file Image_4.TIF]
